# Twenty-seventh Annual Meeting of the United States Veterinary Medical Association

**Published:** 1890-11

**Authors:** Austin Peters

**Affiliations:** Secretary


Secretary Hoskins submitted a report of the Committee on Publication.

President Michener—We will next receive the report of the Special Committee on Centralization.

Dr. Huidekoper—I think your Committee have nothing to report except progress. We have had one or two meetings, but no action has been taken sufficiently definite to submit to the association in the form of a report.

President Michener—The next in order will be the report of the Special Committee on Tuberculosis.

Dr. Hoskins, of this committee, reported as follows:

Mr. President and Gentlemen: I had expected to find in Chicago to-day a full report from Dr. McLean, but it is not here. I can simply state, in his absence, that we followed instructions and carried the resolutions to Washington, where we were kindly received by the Hon. Jeremiah Rusk, Secretary of Agriculture, who gave us a very patient hearing. We found him in entire accord and sympathy with the spirit of the resolution, and willing to aid us all he could to forward the object set forth in the resolutions. I am sorry a more extensive report is not present—it must be in Chicago somewhere.

Secretary Hoskins submitted his report as follows:

I have to report that letters and telegrams of regret have been received from several members.

I have also to report to you the resignation of Dr. A. L. Hummel.

I have also, Mr. President, to report to you the deaths of Dr. G. A. Lathrop, whom we elected one year ago at Brooklyn; Dr. Alexander Lockhart and Samuel R. Percy, honorary members of this association.

On motion of Dr. Faust, seconded by Dr. Hoskins, the resignation of Dr. Hummel was accepted.

On motion of Secretary Hoskins the Chair was authorized to appoint a committeee of three to draft suitable resolutions on the deaths of Drs. Lockhart, Lathrop and Percy, which resolutions are to appear on the minutes of this meeting, and an engrossed copy of the same forwarded to the families of the deceased members, respectively.

President Michener—Since these deceased members are well known by some of our older associates in the East, I should like to appoint as a committee to draft these resolutions, Drs. Hoskins, J. S. Robertson and Dougherty as that committee, and they will act.

### Reports from Assistant (State and Foreign) Secretaries.

On the call of the roll of States, the following responded:

Connecticut, by a communication from Dr. George Bridges.

Illinois, by the reading of a communication from Dr. R. J. Withers.

Massachusetts, by the reading of a communication from Dr. Charles S. Lyman.

Minnesota, by the reading of a communication from Charles I. Heath and a statement from Dr. Lyford.

New York, by the reading of two communications from Dr. Liautard. Also the reading of a communication from Dr. Harry D. Gill.

Pennsylvania, by the reading of a communication from Dr. John Marshall.

Toronto, Canada, by the reading of a communication from E. N. Sweetapple; also by the reading of a communication from Dr. Andrew Smith.

Montreal, Canada, by the reading of a communication from Dr. D. McEachran, Dean of McGill University.

Dr. Lyford, Resident State Secretary, reports: I had expected here an extensive report from the State Board of Health of Minnesota, but it has not arrived. Last year we had sixty-five cases of tuberculosis in our own neighborhood, but I think it will fall short of fifty this year, though within the last six weeks I have ordered twelve cases killed, and two were ordered killed the first of this month.

Wisconsin, Dr. Atkinson reported as follows:

As State Secretary from Wisconsin I do not know as I fully appreciate what you desire. I do not know that I am competent to make an exhaustive report at this time. In this connection, however, there may be a matter of interest to some. My predecessor was Dr. Rowland, of Monroe; he is probably dead by this time. I mention this as a matter of information to the members of the association.

So far as our State is concerned, as I said in my letter, I think the veterinary profession is gradually assuming the place it is entitled to hold. During the last year, as State Veterinarian, I disposed of sixty cases of glanders. We have had no case of pleuro-pneumonia. We have had occasional outbreaks of hog cholera. Our loss from that source is not great, perhaps owing to the local quarantine and the power to enforce it conferred on our Board of Health.

The outbreak of hydrophobia that is mentioned in Dr. Hewitt's report, I think was on the Mississippi River near our State border. A similar outbreak occurred on our side of the river at about the same time, and some six or eight animals were destroyed, although I do not think the diagnosis was verified by any one competent to make it.

We had an outbreak of trichinosis and one death from it in the city of Oshkosh. Trichinosis and tuberculosis prevail to some extent, though not so much in our State as in some others. I am sorry I cannot make a more complete report, and I make this from memory in order that the State may not be allowed to pass.

Secretary Hoskins—Mr. President and Gentlemen: I have applications from J. F. Ryan, W. H. McKinney and J. D. Donnelly for membership, all of whom are properly vouched for.

A communication from Dr. Cooper Curtice, of Washington, D. C., which was also read.

I would like to call your attention to one thing which was omitted, but which can now be done by unanimous vote, and that was the action of the association electing to honorary membership the candidates recommended by the Comitia Minora. I would therefore move that the recommendations made by the Comitia Minora, that the names presented for honorary membership in this association be approved, and that they be duly elected as honorary members. Seconded.

On the ballot being cast, the following named were declared duly elected to honorary membership in the association.[1]

Secretary Hoskins—Inasmuch as we have lost a great deal of time this morning by reason of the failure of the Comitia Minora to get to work at the appointed time, I move you that the discussion of the reports be postponed until to-morrow morning, and that we now proceed to the election of officers.

The motion of Secretary Hoskins to proceed to the election of officers having been duly seconded, was carried.

Dr. Winchester nominated for president R. S. Huidekoper, of Philadelphia.

On motion to close the nominations for president, on a rising vote there were twenty-two for and eighteen against.

Dr. W. L. Williams, of Illinois, was nominated by acclamation as vice-president of the association, and the nominations for this office were declared closed.

Dr. W. Horace Hoskins was nominated by acclamation as secretary of the association for the ensuing year.

Secretary Hoskins—Mr. President and Gentlemen: I have served you now for two years, and the work has grown each year more laborious and important. My professional work at home has also increased, so that I do not feel that I can any longer give the time to the work of the association which it should receive. I would decline with thanks the office which you tender me in favor of anybody else.

Cries of Question! Question!

The nomination for secretary was declared closed, Dr. Hoskins being unanimously nominated for that office.

James L. Robertson was nominated by acclamation to the office of treasurer.

On motion of Dr. Winchester, duly seconded, the Secretary was directed to cast the ballot of the association for the election of the officers who have just been nominated.

Secretary Hoskins—In accordance with the authority vested in me I have cast the ballot of this association, and the following have been duly elected as officers of the association for the ensuing year:

For President, Dr. R. S. Huidekoper, of Philadelphia, Pa ; for vice-president, Dr. W. L. Williams, of Bloomington, Ill.; for secretary, Dr. W. Horace Hoskins, of Philadelphia, Pa.; for treasurer, Mr. James L. Robertson, of New York.

President Michener—Gentlemen, it gives me great pleasure to announce to you that you have chosen your officers for the ensuing year, and that you have re-elected to the office of president, Dr. Huidekoper, whom we all know has served the association well in the past, whom we are sure has the best interests of the association at heart, and that he is a man who will probably best represent the association during the coming year.

[1] Published in October number JOURNAL OF COMPARATIVE MEDICINE AND VETERINARY ARCHIVES.

I thank you very kindly, gentlemen, for having conferred upon me the honor of being your presiding officer during the past year, and I now very gladly resign in favor of Dr. Huidekoper, who will take the Chair.

President Huidekoper—Gentlemen: I certainly thank you for the compliment of my election to this chair. After the address of welcome by Mr. Williams this afternoon, President Mitchener, in responding to Mr. Williams, said that we had just completed the foundation of the U. S. Veterinary Medical Association. This association is to-day holding its 27th annual meeting. True, it is a very old association, but as associations grow, its progress has not been too slow. For a long time it was to a great extent a local organization. The first meetings were held in New York; at that time we held two meetings annually. It is only within the last few years that we commenced to go to other cities than Boston or New York. Then we held a meeting at Philadelphia and one in Baltimore. At an early period there was a meeting held at Washington, and a meeting held in Philadelphia, which, however, were accidental. The first meeting attempted to be held in what we sometimes call the West was convened at Cincinnati, but for various reasons was not a success. Very few men came to the meeting, and none from the West beyond those doing business in Cincinnati. So this meeting in Chicago is really the first effort that we have made to hold a general meeting in the West. It is only within a short time that we have appreciated the growth of the profession in the West. A few of us were some years ago in attendance upon the Cattle Convention here in Chicago where we met a number of prominent western veterinarians. Many of the best veterinarians in the East have come here, so that to-day, in meeting in the West, we may say we have finished the groundwork, the foundation which we have been building for the last twenty-five years, of the U. S. Veterinary Medical Association. We are an organized body to-day, we have taken in some forty members, and this meeting makes it, not only in name, but absolutely in practice, the United States Veterinary Medical Association. I am sure from the correspondence I have seen in the hands of the Secretary that he has done an immense amount of work, and has more before him in the future than that he has so well performed in the past. I think if you could realize the amount of work that has been done in arranging for this meeting you would agree with me that we owe him our gratitude. Every man who comes into this association is a practical man, and cannot but be a benefit to the association. The young men who have come with us to-day will stimulate the older members to more active work, and in this way the association should be very successful.

I think there are several things which every member can do to further the general interests of the association, one of which is to aid in the strengthening of the local organization. In every State an effort should be made to support the local associations, and keep an active society as auxiliary to this association. A few days ago I attended our State Association meeting at Lancaster, Pa. I made a proposition there that the State association undertake the formation of local societies throughout the State, wherever they have four or more veterinarians in a county, and with these organize a county society. We then made a stipulation, to be voted on six months from now, that wherever four veterinarians form a local society, that no one is eligible to the State association unless a member of the county society. If a man is not fit for professional or personal or other reasons to be associated with practitioners in his own county, he is not desirable as a member of the State association. I will not take any more of your time. I will give you my best efforts for the success of the association during the coming year. I think each member can do a great deal to advance the interest in meetings by preparing reports on matters of special interest. It should also be the interest of each member to bring in other reputable veterinarians and strengthen the association in every way possible.

On motion of Dr. Howe, duly seconded, the meeting adjourned to meet at 9 A.M., September 17th, 1890.

WEDNESDAY, SEPTEMBER 17TH, 1890.

The meeting was called to order at 10 A.M. by President Huidekoper, who announced the appointment of the Board of Censors and standing committees,[1] and then said, the next order of business is the discussion of committee reports. The first in order will be the discussion of the report of the committee on intelligence and education. As it appears no one cares to discuss that report, I will call upon Dr. Dougherty to submit the report of the finance committee.

Dr. William Dougherty, chairman of the finance committee, submitted his report as follows: We examined the Secretary's papers and vouchers last night, and submit the following report:

Expenses during the year, $544.69, made up of the following items:

| | | |
|---|---|---|
| Secretary's expenses, postage, express, etc., | $ 71.34 | |
| Appropriation and deficit, 26th annual dinner, | 92.00 | |
| Committee expenses, | 121.60 | |
| Committee of Arrangements, Chicago meeting, | 16.50 | |
| Secretary's salary, one year, | 100.00 | |
| Stenographer's charges, Brooklyn meeting, | 15.00 | |
| Printing expenses, Publication Committee, | 121.50 | |
| To insertion 27 names in certificates, | 6.75 | |
| | | $544.69 |

Collection of initiation fees and dues for year per statement, $581.50. Balance due by treasurer, $36.81.

(Signed) WILLIAM DOUGHERTY,
W. L. WILLIAMS.

President Huidekoper—Gentlemen, you have heard the report of the finance committee, and if there is no objection the report will be received and approved as read. There being no objection it was so ordered.

The next subject for discussion is the report of the committee on diseases.

Dr. Paquin—Mr. Chairman and Gentlemen: It may be that I have not the right conception of what ought to constitute the report of this committee. I had thought that one of its chief elements, at least, ought to be justice;

[1] Published in the October number of the JOURNAL OF COMPARATIVE MEDICINE AND VETERINARY ARCHIVES.

it ought to deal fairly with all the questions it attempts to consider. If it mentions anybody connected with any work, it ought to mention them all equally, or at least, tell the whole truth about it. The report that has been submitted to us on contagious diseases, or diseases rather, consists chiefly in explanations of what one gentleman has done in investigating Texas fever. A large portion of the many pages in the report refer to the good work done by Dr. Smith at Washington. Every strong point is stated with fairness and exactness, but when it comes to compare that with the work of other men, the report glances over the other work, omits entirely much that is worthy of consideration, and then makes an unfair comparison. I submit, gentlemen, if a comparison is to be made, it ought to be impartial. If there is to be merely a comparison of these investigations of Texas fever, they ought to be full and complete of all the work that has been done, and not the opinion of the gentlemen who framed the report. I do not understand this association to be organized for the purpose of discussing one-sided views. I do not understand that reports ought to be based on opinions, but on facts. If I am correct in my opinion, this report should have presented all the facts that have been developed by investigation. I do not object to what has been said concerning the important work of Dr. Smith. It is very important that the investigation he has made be given to this association; but what I do object to is the unfairness with which comparisons have been made. You may, perhaps, pardon me for speaking of myself, but in this connection I presume I will have to blow my own horn. Now, we have made some investigations of Texas fever, and a bulletin has been published. We do not claim it is perfect, for we know there are many imperfections in it, and are satisfied that we will find many more errors, but all these will be corrected. We have attempted, however, to give the facts as we have found them.

Great stress was laid in the report of the Committee on Diseases on the causation of Texas fever, or the germ of Texas fever. The judgment or opinion is based on perhaps half a dozen cases. We, in Missouri, perhaps do not know much about Texas fever, or in Texas, Arkansas or Indian Territory, where we see only a few cases. No men may be as well informed as the gentlemen from Baltimore, where they have seen a few cases in two or three years. But in the southeast we have this advantage, that when we have cases of Texas fever we can diagnose it whether it is produced by inoculation or comes from natural causes. This much is certain, that we know Texas fever when we see it. The gentleman has alluded to our work, simply as our claim: That we have simply claimed things that probably are untrue. I do not claim to say that we have absolutely discovered the germ of Texas fever, whether it is bacteria or the precise form of the germs. We have not classified it. We have simply said there is a micro-organism as the causation. These germs have not been found by myself, originally, but are the same that were seen by Dr. Salmon himself and described by him some years ago. It has been said that our work counted for nothing, because we went to Texas and Arkansas and Indian Territory and there gathered specimens. I want to know if it is impossible to gather specimens in that line entirely perfect and clear of any outside influences? It is just as possible to go to Texas with an alcohol lamp and gather bile perfectly pure as it is in the laboratory which is full of floating microbes. It is a possibility. If it is impossible to do that in this counrry, how is it we can have virus sent from Europe, gathered in the field and not in the laboratory. We cannot have all these cases in the barn. We have got to take them where they are. But suppose it was true that all the work done in Texas, in the field, the specimens sent to us, were all impure, what about those cases we have had under our eyes, and watched from the beginning every step of the disease and gathered specimens as pure as it could be done at once. I do not claim absolute perfection, but we have worked with sincerity and with exactness, desiring to be of service to the medical profession, not working for ourselves or for our own glory. We have been honestly striving to clear some mysteries concerning Texas fever, and not trying to create the impression that we were investigators. But, if we assume that all we have done, so far as the germ itself is concerned, is incorrect, there is still something that should have been noticed if this question was to be considered at all, and that is, some of the questions relating to Texas fever, outside of the germs, outside of the actual causation. I mean to say that there have been points brought out by our investigation that undoubtedly shed more light than ever existed before on this subject of the germ of the disease. The chairman of the Committee on Diseases, as I say, has presented a strong point of the gentlemen who have studied Texas fever in Washington, and has merely given the weak points of our investigation. He has laid much stress upon his opinion that Texas fever in cattle is similar, if not identical, to malaria in man. If he meant by that that Texas fever is just like malaria in man or something like it, I believe he is greatly in error. In the first place, malaria in man is not taken from one State to the other. You can go to the Indian Territory or Missouri, where they have malaria, and then go to the North and our children and famlies do not take it. Texas cattle feeding on infectious ground, taken North, communicate the disease so that native stock exposed to it die from Texas fever. There is certainly this difference so far as the actual nature of the disease is concerned. It may be true that the two germs referred to are somewhat alike, but as to the nature of them and the method of transmission of the disease, there is certainly a great difference. Some statements have been made to this effect which are so vague as to hardly justify a reply. We have made inoculations with as much care as could be done, and there are gentlemen in this room to-day who have seen cases of Texas fever produced by inoculation, from native to native, that have produced death. That is a possibility. I have never known such to occur in malaria. Another point worthy of notice in Texas fever, which was entirely ignored, with a view to casting a reflection on everything we have done is the agency in which the virus is transmitted from the South to the North. The gentleman has omitted to state that in Dr. Smith's work he says: I do not know yet in what agency the virus is transmitted to the North, and we are yet in ignorance of that fact. Farmers in the Southwest have known for years that some agency could bring Texas fever to the country. They have known that urine alone could transmit it. They have known that manure alone could do that, and our experiments have simply confirmed this opinion. We do not claim to be the first in that discovery.

Now, gentlemen, I submit that if this question was to be brought before this association it should be discussed fairly; there should be no partiality. I am not alluding to anybody personally but generally, I consider this report unfair to myself, and therefore have made this explanation.

Dr. A. W. Clement—Mr. President, as chairman of this, committee it has been my endeavor to review the work as impartially as possible from every standpoint. I happen to have been very much interested lately in malarial diseases. From reading Dr. Smith's report it occurred to me that Texas fever might be closely allied to malaria; and in making this report I laid special stress upon those diseases which I thought might be of misamatic origin It was certainly not my intention to be personal in the matter, and I do not think I can justly be accused of such But every man's print is public property and is liable to criticism. In dealing with these things I have gone into them as closely as possible, but it would take the entire time of this meeting if every detail was to be fully considered. I simply remark that from a review of Dr. Smith's work in connection with malarial diseases, I thought that the germ of Texas fever closely resembled that found in malaria. I do not pretend to define the difference, as I have not investigated Texas fever myself to any extent. I have seen very little of it and that from a laboratory standpoint. It was a very disagreeable duty, of course, to criticise the work of Dr. Paquin. I have known him for a long time and have thought a great deal of him and appreciated his work, but when a man puts into print such opinions as he has advanced as chairman of this committee, I could not overlook it. It certainly is not regarded according to the methods of modern bacteriology to collect specimens in the field and have them carried as far as he has done. It is absolutely impossible in the opinions of those who are authority on the subject to prevent the organisms of many varieties. He says in so many words that they have found several organisms in Texas fever, all of which he considers different forms of the same organism. He describes baccilli and rods much longer than they are wide. He describes oval organisms and round organisms and so many varieties which differ essentially from each other that it seems impossible that they should be in any wise related. He says that he has produced Texas fever by inoculating these organisms, but he does not say what organism is. That he produced it by inoculating several organisms. For that reason, I say that his work is not such as to be considered scientific. He says that he has conferred immunity upon cattle by inoculation, but he does not say how he has modified his virus, and in that respect his conclusions are not clear. I do not know of anything more I can say, only that if I have done him an injustice I am sorry, as it was the furthest thing from my mind. As I say, such a work I do not consider to be of any practical value and I do not know how I can answer it any more definitely.

Dr. Paquin—If you will allow me one more word. I did not mean that the gentleman intended any personal injury or anything of the kind. Dr. Clement has said that it is impossible to carry virus without putrefaction. Of course, if you leave it open, it is impossible, but suppose you seal it. Suppose you take gall, or take the bile, and with a sterilized pipe seal it up before it has any chance of contamination. Cannot that be carried without putrefaction? Certainly it can. We have some two or three years old that is still kept without putrefaction. If the material be gathered perfectly pure before contact with the air, and then promptly sealed, it is possible to carry it any distance. And that is the way our material is gathered. This gathering of the material was simply part of our work. We are still experimenting, and if any discoveries are made we will state them. I have not attempted to give any method of modification of the virus, as I have not modified it at all. If a germ of Texas fever is passed through Northern cattle, it will produce fever of the same symptoms as Texas fever, the same effect in the blood corpuscles. It is already modified by nature, and it is not necessary to modify it by any mechanical processes. So it is possible to produce it to some extent, and we can produce immunity for a while simply by virus taken from one of our own cattle suffering from the disease. On the question of immunity I have one more word. If the disease were of the nature of malaria, how could we explain, on that theory, the fact that Southern cattle are immutable against Texas fever? How can we explain the fact that the cattle of Texas, always exposed to the infectious soil, are immuned? By what possible method do they become immuned? How do they gain that immunity? We take Northern cattle to Texas, and within ten days, in the Summer months, they die of the Texas fever. We take Texas cattle to Northern pastures, and in thirty, forty or fifty days in our pastures they die of fever. But you take the calves born on Texas soil, and constantly exposed to this germ, very few die of Texas fever. Take these same cattle North for three months, and return them to the South, and they will take Texas fever and die from it. If that is the fact, these cattle must have had immunity by some method on the Southern soil, which they lost on the Northern soil. After losing it on the Northern soil, they die of Texas fever. That is not an experiment; I have seen it myself time and time again. Now, then, if this immunity is gained, it must be through some kind of virus. I have found that the Southern cows, so far as our experiments go, are somewhat affected, or, at least, contain in their blood, at some time or other, a parasite that can be detected by microscopic examination, as has been explained by Dr. Smith. It may be outside the blood corpuscles; it may be contained in the liver, in the bile of calves born from those cattle, which calves must naturally be vaccinated from their mothers, when they are born on Southern soil, yet they have been able to resist the germ on the Southern soil. It must be that immunity is conferred by principle, and it is on that principle we have been working. We have been trying to find out by what principle immunity is conferred, and if we could find that out, it would solve the whole question of Texas fever, and therefore, gentlemen, we have tried to be honest in this matter. Our methods have not been perfect, perhaps, but we have not lost any specimens by putrefaction.

Dr. Salmon—I think, in justice to the investigations that have been made in the Bureau of Animal Industry—if not from the point of justice, at least from the point of making an examination in regard to our work—that it would be proper for me to say a few words at this time, although I shall cover the subject of Texas fever in a paper I expect to read to-day. I regard it as unfortunate that there should be so much feeling between different investigators in the same field of work. There is room enough for all of us to work, and surely enough to be done, and the important question connected with these diseases will be solved none too soon if we all work and strive together to throw what light we can upon the subject. As far as I am concerned, I feel no jealousy in regard to the work of Dr. Paquin or any other investigator, and I should feel regret if I knew there was any such jealousy in regard to my work, because I appreciate the importance of all working together. We will naturally get some different results, but when we come to put those results together, and study them and draw our conclusion, we will probably see that each from his standpoint, from his methods of work, is able to throw light upon the problem which we all desire so much to understand. The subject of Sexas fever is undoubtedly one of the most difficult problems that investigators in this or any other country have ever attempted to solve. It is a disease peculiar in its characteristics. It is surrounded with something of a mystery which cannot be understood from our knowledge of other diseases. Therefore when we work upon this disease we are, to some extent, in the dark. We have not the light of other investigators to lead us in solving the different questions which come up. Another thing which is important. We claim, in our investigation, that by cultures which we have made in recent years we have been unable to get any growth, any germ whatever, in our culture. Others claim they have been able to get cultures. The methods are somewhat different. It is for those who look over these investigations and study them, who endeavor to understand the nature of the diseases, one worker gets cultures and another cannot. Dr. Paquin spoke of Dr. Smith's work, or Dr. Clement's conclusion from it, rather, and he throws out the insinuation that the material which we have had and studied has been very limited. This is a mistake. For four years we have studied that disease during the whole season in which it exists. We have brought cattle to our experimental station from North Carolina, where the Texas fever is prevalent, and we have put them on our experimental stations, and have produced as many cases as we could possibly study during one season. We have had another advantage over Dr. Paquin, in that we have had more men to help us in this work. We have had several men in the laboratory to keep our cultures, to make our cultures, we have men to make our microscopic examinations, and we have had men at the experimental station to watch the animals and observe symptoms. We have so divided our force as to get as much assistance as possible from the different workers. Therefore I claim we have made as many observations, and as carefully as it was possible to make from any point of work. Now, I want to say a word in regard to germs. Dr. Paquin has been extremely liberal in his remarks in regard to the discovery of the germ of Texas fever, even giving me credit for having discovered the germ years ago, which I described in a report to the Department of Agriculture, back in 1880. That was the form of germ which he also described. Now, I must disclaim any credit for the discovery of this germ, which I described at that time, and it can have no possible connection with the germ which Dr. Smith has described. If you will read my report, you will remember I do not claim that that germ was the cause of Texas fever, and did not at the time I described it. I carried on my work at that time as some of Dr. Paquin's work has been done. I was obliged to work at long distances. I did not have as good a laboratory as I have now. I was obliged to gather my material in the field, and seal it up in tubes and carry it some hundreds of miles to my laboratory, and there work it out. Our work in Washington has been very different. We have had stations close to the laboratory. We have had every facility for communication between station and laboratory. We have had all the material which we could possibly work, so that I think, so far as the observations of our bureau go, they have been carried on under conditions which make the results fully trustworthy, and as reliable as it is possible for such investigations to be. Now, one word more in regard to the germs. I do not consider it possible that the bacteria, the germs which have been described by Dr. Billings and Dr. Paquin and by myself, they certainly were bacteria. I do not consider it possible that those germs can be confused with the micro-organisms which Dr. Smith is working on at present. They are found in different localities. The bacteria are cultivated, and they do not resemble one another in the least. So, so far as I am concerned, I am ready to say those investigations are entirely different, and the germs differ from the germs which Dr. Smith treats of, radically. So I see no reason for any confusion. So we must conclude that the germs discovered by the Bureau of Animal Industry are entirely different from these germs we have been working with.

Now, there is one remark which Dr. Paquin has made which I would like to say a word about. In inoculating from material of dead animals he gets certain results, and in certain cases produces death. Now, the question is, have you produced the disease which you supposed was contemplated, or have you produced a different disease by the peculiar germs which you have inoculated? This is a point which some of us may not have realized. I know in my own early investigations of Texas fever, where I got most decided results by inoculation, that these results were not, as I supposed, Texas fever, but they were the result of inoculating with organisms which produced a malignant type of a different disease. So, if you are going to diagnose diseases to-day of the nature of Texas fever you must know what the germ is, and you must be able to recognize the germ when you see it, without the possibility of confusion with other micro-organisms, and then, when you examine the liquids or the tissues of the affected animals, you can ascertain positively whether your animal is affected with a particular organism or not. So long as there is confusion in regard to the nature of the germ of Texas fever, so long must there be uncertainty in the diagnosis. When we find this peculiar parasite in the red blood corpuscles of animals we feel sure that it is diseased. We have not studied any of the diseases of animals in this country in which there is any such micro-organism found in any such location as that. If there is a germ in the bile, as well as in the blood, if there is a germ found in the tissues, in the nature of bacteria, he is uncertain whether that germ is what I described in 1880, or they are the bacilli or bacteriæ which Dr. Billings discovered, the short bacillus, more lately, or the long bacillus. Then there must be doubt in regard to the diagnosis of the disease, no matter how carefully investigations are made At present when we diagnose a case of Texas fever there are two points we take into consideration, and we feel sure if we find those points that we are working with one and the same disease: One is the presence of this germ in the red blood corpuscles, and the other is the rapid diminution of the corpuscles themselves. Dr. Paquin is certain, he says, that he can diagnose Texas fever, but he does not tell us how he is certain. He does not describe definitely and distinctly the germ which he asserts he finds in one of the animals affected with Texas fever, and in the young calves as soon as they are born, or before they are born, from Texas mothers. I think that is an important point which Dr. Paquin should have brought out clearly and distinctly if he expects any scientists to accept conclusions of so much importance and so far-reaching as those which he has set forth in his report.

Dr. Paquin also tries to point out weak spots in the conclusion of Dr. Smith that Texas fever is a disease of the nature of malaria. One of his points is that malaria is not transmissible from one point of the country to another, while Texas fever is. Now, I don't think any conclusion should be reached from two facts of that nature. In the first place, a bacterial disease of one kind may be carried to another part of the country, and spread among animals of the same species, while another bacterial disease may be carried with greater difficulty, if at all, so that one disease may be carried, and another, closely allied, might not be carried. And we must consider the fact that there is a great difference in the exposure of people as with cattle. We can expose northern cattle to southern cattle if there is simply a little difference between them, and in a great majority of cases they do not take Texas fever. People are exposed to each other in somewhat the same way. Cattle have another method of contracting infection which people do not have. Cattle go upon pastures, and they eat food from the ground which has been soiled by the excretions of southern cattle. There is no possibility of people contracting the germ in this way. So I think that facts of that kind simply support Dr. Smith's conclusion. He does not claim, and I do not claim, that the diseases are identical, but that the micro-organisms which cause Texas fever are closely allied to those found in malaria. Now, just another point to which I ask your attention for a moment. Dr. Paquin says Dr. Smith is not certain how the disease is conveyed from southern to northern animals. Dr. Smith states this in his report. He says the farmers of the country are certain how the disease is transmitted from one animal to the other; that they know that the germs are, and that they may be, transmitted by the manure and urine. I doubt if we can accept such a conclusion as that. How do they know it when those who have investigated the subject are not able to tell certainly. I do not believe any man, I do not believe Dr. Paquin, can get up here and say he is sure that the disease is transmitted to southern cattle though the medium of the manure scattered on the pastures. I do not believe that he can point to any positive evidence where Texas fever is spread by means of the urine and manure cast upon the soil. That question is obscure, upon which we have no definite evidence that I have seen, and which I think we can very well leave to be decided by investigation in the future rather than jump at conclusions before we have evidence on hand to justify them.

Dr. Paquin—Just another word. I am glad to see the spirit of discussion, as it is exactly what I wanted. I will reply to Dr. Salmon's last question by stating two experiments, or two series of experiments, with urine and with manure. Pens of about twelve feet square and six feet high, where there had been no Texas at any time, were prepared. On the grass of those pens was spread manure in some of them and infected urine in the other. We exposed northern cattle to that grass, and we produced Texas fever. We had not only one case, but several cases from the two different pens, and from the two different kinds of infection.

Dr. Salmon—Let me interrupt you just a moment. What the Doctor stated before was that the farmers of Missouri had been perfectly certain for years that the disease was produced by manure and urine.

Dr. Paquin—I said that they were sure there was some method of transmission. As to the inoculation and the diagnosis of the disease, I beg to say this: That it is impossible to take a spleen or a liver of a diseased animal suffering from Texas fever, make a fluid of it and inoculate another Northern animal and produce the disease, and in the blood corpuscles you will find the organisms as Dr. Smith has stated.

Dr. Clement—I would like to say a word. The basis upon which I criticized Dr. Paquin's work, as I said before, is the fact that he found so many germs which he considers as the cause of Texas fever. There must be one germ. You cannot have Texas fever caused by several different organisms. Dr. Paquin says: "Some of these bodies appear almost spherically, others like bright specks and others like oval bodies." He says the germs were found in the blood and bile frequently. Now, he says this blood can be taken and carried perfectly pure. The fact that he found so many different germs in his blood and bile is very conclusive evidence that it cannot be carried perfectly pure.

Dr. Paquin—Do I say so many different germs, or different forms during the growth?

Dr. Clement—In another place you say you classify them.

Dr. Paquin—I do not say I classify the germs.

Dr. Clement—You cannot have a disease producing virus of several different forms and call one bacillus and leave the rest unclassified. That is the basis upon which I made my criticism.

Dr. Salmon—Just one word. I do not know that Dr. Clement has any means of knowing exactly what Dr. Paquin has seen, except from his report. It may be Dr. Paquin is unfortunate in the way his report is written. Certainly, from reading that report, I can find nothing that would lead me to suppose that the Doctor ever saw a micro-organism in a blood corpuscle, such as Dr. Smith described. He does speak of its being found, but he does not give a description of it. He speaks of it being an oval germ. So, from reading his report, the only conclusion I could reach was that the germ which he studied was bacteria. I do not see how it would be possible for him to study this germ of ours in the red blood corpuscle. It is only by the peculiar appearance of this germ in the red blood corpuscle that we are able positively to distinguish it. After reading this report of Dr. Paquin's, I certainly reached very definite and positive conclusions in my own mind as to what he had seen. I do not see how it is possible for him to have studied the germ which exhists only in the red corpuscles. I do not see how it is possible that he should have described the germ which is found also in the bile and other places where there are no red blood corpuscles. We only pretend to recognize the germ when we find it in the red corpuscles.

Dr. Griffin—If I inoculate an animal with the germ and it gets a disease, would it be in the urine?

Dr. Salmon—It is a question of the recognition of the germ. Without a description of the germ, we do not know what investigators have been working at.

Dr. Adair—If the germ that causes the disease is only in the red corpuscles, and not in the urine, and you inoculate with the urine and produce Texas fever, and the post-mortem shows the symptoms, do I understand the germ would be discovered and the disease diagnosed by the red blood corpuscle?

Dr. Salmon—What I said was I could not undertake to recognize the germ outside of the red blood corpuscle because of the peculiar characteristics of it. We think it cannot be seen outside of it. I do not say it is not in the urine and in the bile, but I do say that the gentlemen who have studied it in the West have not given us any definite description of it so that we could recognize it outside of the blood corpuscles. It may be that they have produced the disease in the way they say, but I question the diagnosis. How do you know? What were the germs which you produced? Give us a definite description of them, and then we will accept your conclusion. Until you do this, we must proceed to further investigation, and at least conclude that the subject is in doubt.

Dr. Hawkins—Before closing that discussion, I would like to know whether the germ of this disease is destroyed entirely by the cold weather? Several years ago, while practicing in Canada, we had Texas fever in a herd that was brought in there by some cattle that had been unloaded on account of an accident. The next season we had a breaking out of Texas fever on the same farm, but not having any Texas cattle, or any that had come in contact with Texas cattle on the place that season, it seemed strange. I have understood that the cold weather would destroy this germ.

Dr. Adair—What time of the year was this accident?

Dr. Hawkins—The latter part of August, or the fore part of September. The fever broke out the next year about the same time.

Dr. Winchester—If the subject of Texas fever has been thoroughly discussed, I would like to make a statement in regard to tuberculosis. If my memory serves me right, at the conference at Berlin last month, Dr. Koch made a statement that he thought he had found something that controlled the action of the bacillus and tuberculosis in animals. That something was written and put in a sealed letter and given to the Academy of Medicine twelve months before Koch made that statement, by two Frenchmen whose names I do not recall. I only make the statement to show that the same investigation had been held and the same results produced twelve months previous to Koch's making his statement in regard to that held in abeyance the activity of the virus.

Dr. Liautard—I may add to that statement made by Dr. Winchester, at in the Academy of Medicine in Paris, they claim by inoculation they had so far obtained these results that animals which had been inoculated with the strongest virus of tuberculosis had been kept after the inoculation for a number of months free from the disease, while others which were vac cinated, as you say, had died in a very short time.

Dr. Salmon—I think the two men were working on entirely different subjects. It seems to me from what I have seen of the article, although they are both written in such a way as to mystify, that Dr. Koch had been working in one direction, and the French have been working with some substance to produce immunity. I judge that they had been working with some materials in which the bacilli tuberculosis had grown, while Koch states in his paper that he has discovered a substance which, given to the animal, prevents the further growth of the germ in its tissues. All the indications are that these investigators have been working on different lines. Most of them have failed in producing what they claim to at present, but their investigations will be of benefit to the medical world.

Dr. Clement—In quoting from the article, I simply made the statement that Dr. Koch said he had found the substance. He did not describe it, and he was not prepared to say just at present what it was.

President Huidekoper—The next subject for discussion is the report of the Special College Committee.

Dr. Lyford—I will say that I have received another letter from the New York College, in which they report progress. I might say that I have received letters from all the colleges except the Chicago College, and each of them, so far, are not only willing but apparently glad to enter into the three years' course, and none of them find fault with the preliminary examination. Thus far the reports are quite encouraging. I have not made a report of the agricultural schools, or consulted them, as I thought that would be of secondary consideration, and as most of them are hardly prepared for such a course. I have thought if we first succeeded with the regular veterinary colleges, we could probably work the others in easier. I should be very glad to hear from the other schools; and as we have Prof. Liautard's report, and as he himself is present, we will probably get some new idea from him as to his work in this line.

Dr. Liautard—Mr. President and Gentlemen: This is a very important question. The fact that there has been brought before the association for a number of years this subject, shows that it is important. It has made some progress; although it has been slow, undoubtedly a good deal will be developed in time. There is no doubt but that a long course of study is a necessity, and that it will impose itself as veterinary science progresses in this country. I will say this, that the requirements for admission, matriculation, etc., were initiated by the American Veterinary College, being the first institution in this country which required it. From the moment this institution was opened we required a matriculation examination, because we felt that our men are engaged in scientific professional work. It was necessary that we should have everything as clean as possible. What was the condition of matriculation previous to 1875 in the veterinary school? Why, a gentleman came in, paid his fee, looked at us for a short while, unable to follow our lectures, unable to follow the progress we were making, unable to take notes, or perhaps in some cases unable to write them. He remained a little while, went away and concluded we had robbed him of his fee. We could not refuse him. He had complied with the requirements, merely paying his fee and yet we knew we were not doing justice to that class of men. Then we started this matriculation examination as I say, the first one I believe in this country in a veterinary college, and I am strongly inclined to believe—you know in 1875 we started that by a sort of examination, so that the student might be able to appreciate the course of instruction and be benefited by it. This preliminary examination is simple; it is not probably what it ought to be. It is not yet up to the standard of some of the other schools, but probably will come there.

As to the question of a three years' course, I believe we are to consider the fact that at first there was not a veterinary high school in this country. The most important point was to try and displace the ignorant man with good practitioners and give these men a good foundation—what might be called a veterinary A, B, C for their own personal benefit. It is for this reason that the American Veterinary College stuck to those requirements. We have inaugurated a change as you have been told by Dr. Lyford. We have lengthened our course from four and a half to six months. We have not yet reached the three years' session at college, but we have required a more lengthened course of study. The day will come, I believe, when it will not be a question of ordinary attendance in school, but rather one of the thorough education of those students who are to come to our schools. I can say as a result that the students in our colleges to-day are better educated than those in the past. The students feel that by a long course of lectures they are benefited by it and rendered competent for the discharge of practical duties.

As to the question of examination it is one that will have to be considered by and by. I am certainly in favor of an examination by each individual school. Already, as I wrote Dr. Lyford, although he has probably not received my letter yet, I have considered the subject of a general board of examiners as a very important step. I have considered the subject also in some of our meetings, and I am thoroughly in favor of a Board of Examiners, but I must say if we are to gain any benefit by a Board of Examiners, we must so arrange it that the individual school would not be tempted in the matter, and, therefore, if that Board of Examiners is to be established, it should be under the control of this National Association, representing every part of the country, and in this way I think we could accomplish much good. Let your examinations be careful and impartial of every one of the candidates whether from the Eastern or Western colleges. (Applause).

President Huidekoper—We will now take up the discussion of the report of the Committee on Army Legislation.

Dr. Griffin—Mr. President, I take this opportunity on behalf of the majority of the army veterinarians to thank the committee for their work in our behalf. The report mentions the fact that the plan meets with opposition from members within the service. This must be expected where we have three men who are not graduates of veterinary colleges, and who upon the passage of a bill creating the corps, and compelling the members of the present force to pass an examination, would be thrown out of their employment. Those three men are strongly opposed to any reform. I am sorry to say, also, that there is at least one other man who is a graduate of a very reputable veterinary college, graduating some ten years ago, and supposed to be a man well educated, yet who fears that in case of an examination he would not be able to pass. This man is exerting all his influence to obstruct progress of your committee on army legislation. There are only fourteen of us altogether in the service, and out of this number I believe there are about six who would fail in the examination, and those men, of course, oppose any bill that tends to reform the service.

The inducements for entering the army are not great, which is the reason why we have such a poor class of men in it. This is the state of affairs and shows you where the opposition comes from. I think it is certain that the competent men in the service who are not afraid of an examination, will lend all their aid in support of the action of your committee; and, as I remarked, it is on behalf of these men that I tendered the thanks of the majority for the efforts of your committee on our behalf.

President Huidekoper—We have present with us Dr. Schwartzkopff of the Minnesota University, who with your permission will present a paper. We also have some other papers sent here, which will be embodied in the report if there is no objection.

We will now receive the paper of Dr. D. E. Salmon.

Dr. D. E. Salmon, Chief of the Bureau of Animal Industry, Washington, D. C., presented his paper on "Some of the last studies on Bacteriology, as it refers to the domesticated animals and the diseases in them."[1]

The paper of Dr. Salmon was illustrated by numerous enlarged views of different germs cast upon the canvas, the diameters of the germs being multiplied thirty thousand times, and their peculiarities pointed out and explained in detail by Dr. Salmon during the course of his lecture. Among others was a specimen from gelatine culture one day old; hog cholera germs. the same germs from a gelatine culture fifteen days old; the same germ from a liquid culture one day old; a germ described by scientific men as the swine plague germ; the swine plague germ obtained by inoculation; a hog cholera germ, with an original stain; a preparation from an animal infected with Texas fever; a preparation of hog blood; a preparation made from blood before the death of the animal, showing double germs of diamond shape, found in Texas fever and malaria; a preparation made from the kidney of an affected animal; a preparation from the kidney of an affected animal, showing the same thing with larger germs, and showing where the germs are to be found; a preparation made from the spleen, from a different animal; an affected blood corpuscle, showing the different forms of germs, with two pear-shaped parasites in it.

Dr. Salmon also exhibited views of several places where the board had had difficulty in ridding the locality of pleuro-pneumonia, showing adjacent pools of stagnant water, dilapidated buildings in Brooklyn, in some cases where the pool of water had its course under the buildings. Pleuro-pneumonia could only be obliterated in such a case by the utter destruction of the

[1]Published in the October number of this Journal.

buildings, the digging up of the ground and a thorough disinfection of the soil.

President Huidekoper—I think, gentlemen, you will all agree that Dr. Salmon's paper is too important to be discussed hastily, and therefore a motion to adjourn will be in order.

On motion, duly seconded, the association adjourned, to meet at 1.30 P.M.

2 P. M., SEPTEMBER 17TH, 1890.

The meeting was called to order, with President Huidekoper in the chair.

Vice-President Williams took the chair, and Dr. Huidekoper read a paper on "The Contraction of the Horse's Foot and Contracted Heels."[1]

Prof. A. Liautard, dean of the American Veterinary College, then presented his paper on the subject of "Veterinary Jurisprudence."[2]

President Huidekoper introduced Dr. Olof Schwartzkopff, professor of veterinary medicine in the University of Minnesota, who read a paper on the subject of "National and International Meat Inspection," as follows:[3]

Dr. Burns, of Brooklyn, was introduced by President Huidekoper, and read a paper on the subject of "Moss as a Substitute for Linseed Oil and Hot Woolen Cloths, as follows:[4]

President Huidekoper introduced Dr. J. C. Meyer, Sr., of Cincinnati, Ohio, who read a paper on the subject of "Cotton-Seed Oil Cakes."[5]

Dr. Meyer, Sr.—The remarks I have to make on Dr. Salmon's paper are simply to say that I was very much pleased with it. But I would like to know what he has to say about his statement that these organisms are in the animal's body, and that there are also developments going on in the outer world. I would like to know how it is about the germs being developed partly in the animal's body and partly on the outside.

Dr. Salmon—I do not know that I can add very much to what I have already said. What we do not know about Texas fever germs would make a very much larger book than what we do know. I have said all we would like to say about it. We do not know anything about the life of the germ outside of the body; we have not been able to recognize it. I do not know where it grows or how it grows. We simply know that when a pasture is infected with these germs, the infection seems to be intensified as the season goes along, and I know that the cattle take the germs more rapidly into their system later in the season than earlier in the season. What form they assume, and how they grow and what are the conditions necessary to their growth, or how they are taken into the system, we are still in ignorance. Those are questions it will take several years to solve.

Dr. Meyer, Sr.—In respect to lame horses, I think we would do well to send them to the Chicago Sanitarium. I have seen so few lame horses here. I guess it is because the streets are so level that there is little occasion for spraining and the like.

[1] Published in the October number of this Journal.
[2] Published in the October number of this Journal.
[3] Page 599 of this Journal.
[4] Published in the October number of this Journal.
[5] Published in the October number of this Journal.

Chairman Hoskins—If there are no further remarks on that paper, we will pass to the paper of Dr. Liautard, on "Veterinary Jurisprudence." I hope that the question of veterinary jurisprudence has not been so thoroughly exhausted that there are no remarks on that subject from as many States as we have represented here to-day. In some of them it must be that judicial decisions have already been rendered which will add interest to the consideration of this subject.

President Huidekoper—If no one has anything to say, I would like to remark that it is a matter which should be thoroughly gone ever, and brought up again at the next meeting. I have had some experience myself with the Continental system, which Dr. Liautard regards as better than our English system, based on the words "soundness" and "unsoundness." It is one that certainly is of great interest both to the purchaser and the seller. There is one point which Dr. Liautard did not bring out, and that is the Continental system, with its redhibitory vices, which the law charges the seller with, unless he is specifically relieved therefrom by the written contract of the buyer, in nine or thirty days, as the case may be. Then, too, the law assumes to cover only the hidden vices. All open troubles—spavin, ringbone or anything of that kind which can be seen by an ordinary expert--they assume that the buyer ought to see, and if he is not sufficiently expert, then he should employ the proper one to make the examination for him. That allows the veterinarian to be, in turn, a great deal more lenient with the dealer. It allows the veterinarian to recommend the purchase of a horse with a visible blemish, which will not injure him, as in the case of an anchylosed spavin or ringbone, which allows a horse to do a hard day's work without a lame step. It allows the sale of such an animal, with the purchaser having a knowledge of that. Where an ordinary certificate uses the words sound and unsound, you would be compelled to condemn a perfectly useful horse having such a blemish. I think, with a little unanimity on the part of the profession, it would be comparatively easy to obtain suitable legislation looking toward a change in the system of examination. Personally, I find it entirely practicable, in many examinations, to carry it out by the permission of the seller and buyer. In several dealers' stables in Philadelphia where I practice, examinations are made in that way, and I do not use the words soundness and unsoundness as they occur on my ordinary certificate, but I scratch them off the paper and state the blemishes of the horse, and whether I consider he will be useful or otherwise. I would like to hear the subject discussed.

Dr. Atkinson—I would like to inquire of Professor Liautard whether the Continental law he speaks of is statutory or common law?

Dr. Liautard—They are special laws for that purpose. I will interpolate into my paper that in regard to redhibitory vices, they are referred to, so long as the animal was warranted not being free from them.

Now, I do not know that a great deal of discussion can be had on this subject, for the simple reason that it is so broad. It is an important step to ask that we should work for the establishment of laws different from those which have been in existence in courts for many years. It may seem presumptuous on my part to present the subject to you, yet, at the same time, what I have said is the result of many hours of thought, and I have the regret of knowing that in many cases, by rejecting the animal under the law, we are doing a great injustice to all parties concerned, and that those horses which we were obliged to condemn under the law would be perfectly satisfactory, and give good, useful service to the buyer. Now, that subject seems to me of great importance. We cannot discuss it fully, because there are so many points to be considered, It seems to me, as our President has suggested, that we should not allow the paper to go by—the paper may go by, but for pity's sake, do not allow the subject to go by. It is of as much importance as the paper read by Dr. Schwartzkopff; it is a national subject belonging to veterinary science, and we ought to take hold of it. I think it should be kept before the association.

Dr. Atkinson—I do not know that I fully understand exactly what is meant. I have given the subject some thought, and it seems to me that a uniform law in this direction would be difficult to obtain, owing to the peculiarities of our form of government. Laws, as I understand them, come from two sources; there is statutory and common law. Common law is the decisions of the courts that have been handed down from time to time, and are based on the ideas of justice that obtained at one time or another, some of them coming to us away back from the time when kings had the power to issue edicts. Statutory law is the enactment of the different State legislatures and National Congress. Enactments generally are the result of some emergency which the common law does not provide for. Unless they are the result of such an emergency, the statute law is liable to fall into disuse. Now, under our Constitution, I believe it would be impossible for the National Government to enact any law in relation to contracts and their enforcemənt between citizens of the same State. So that any uniform practice would have to be established by the statutory enactment of the respective States. As it is now, the decisions have nearly all been rendered under actions for what is known as breach of warranty, implied or expressed, implied by some statement that the seller had made to the purchaser verbally or in writing. In proceeding under a breach of warranty it becomes necessary to establish that the warrant was expressed by the seller and was acted on in good faith by the purchaser, and that the animal was not up to the representations made. Uuder our State law (Wisconsin), and I presume it is the same in other places, the purchaser would have the right to proceed in one of two ways upon a breach of warranty, either return the animal or thing purchased and demand full return of the purchase-money, or retain it and sue for the difference between the real value and the value which he paid. Now, in applying the law, we want to make it suit all cases. If there is special legislation that touches it; for instance, if a man sold an animal without warrant, we will say a horse that was sick, that proved to be affected with glanders, under our law he would not need to establish that it was represented to be sound at the time it was purchased. A special act of the legislature makes it unlawful to sell such an animal, and the purchaser may recover the price paid and other damages.

I do not know how uniform legislation could be had under our present State constitution. I have ventured thus far to express my views on the subject, although they may not throw much light on the discussion.

President Huidekoper—The Chair would like to answer one of Dr. Atkinson's propositions. Fortunately, in the West, most of the States have laws concerning contagious diseases. Take the illustration he has just made of glanders. But during last Spring in Philadelphia a case was brought into court of a glandered mare that had passed through the hands, during the period of two years, of some six different purchasers. Each one in turn had sold the animal with full knowledge that she was glandered, several of them knowing that they and their neighbors had lost horses through contact with this animal. It was stated by the judge in the Pennsylvania court this Spring that there was no law in Pennsylvania that forbids the sale of a glandered animal, so that for Eastern States we need additional legislation very decidedly.

Dr. Atkinson—That covers exactly the point I had in view, that where the common law is not sufficient to meet emergencies, statutory law must be enacted. In our State it is a criminal offense to sell an animal affected with such a disease, and the seller is liable to criminal prosecution, fine and imprisonment. That is a case where the statute is in aid of the common law, and supplies the deficiency. Under the common law the seller would be liable for a breach of warranty or failure of consideration of the contract.

President Huidekoper—Remarks are now in order on Dr. Schwartkopff's paper on the Meat Inspection Law. He has presented a very important subject, and I would like to hear from some of you State inspectors on the subject. Dr. Salmon, will you give us something on the question of meat inspection, as you have just been doing some national meat inspection?

Dr. Salmon—If there is any light I can throw on the subject I would be happy to do so; but I hardly know what points the gentlemen are interested in. If any care to ask me any questions I will be glad to tell them anything I know about it.

Dr. A. H. Baker—I would like to ask what is the status of the meat inspection of American cattle in England?

Dr. Salmon—I found that to be rather peculiar in certain respects. There are a large number of cattle landed at three or four different places where they have large cattle sheds and docks, and suitable accommodations for the cattle; but they have but one inspector at each place where there are sometimes a thousand cattle landed in a day. The Inspector stands on the dock and looks at the cattle as they come in from the ship, and if he sees anything the matter with them as they pass such cattle are run off into a pen by themselves for future inspection. That is about the end of professional inspection, although I think there is an arrangement between the inspectors and the butchers that in case any marked defects are found they are to be reported. If there is such an arrangement, it is more or less *sub rosa*, and I do not know to what extent it can be relied upon. It struck me that the inspection made over there of our cattle by English inspectors was not very thorough, although perhaps as critical as we would care to have it made.

Of course, as you know, the Department of Agriculture has succeeded in making arrangements with the British Government, by which we have placed three inspectors over there, one at London, one at Liverpool, and one at Glasgow. They are there of course only by the courtesy of the British

Government; we have no right to send men there to make an inspection on British soil; but it was done by the Government because there had been a great many reports of pleuro-pneumonia being found among American cattle on the other side. Some of these reports were made in regard to cattle shipped from parts of the country where we had no evidence of there being any contagious pleuro-pneumonia, and it seemed very desirable that our Government should have these men there to look after the matter for two reasons: First, there was a possibility that there had been an error in diagnosis. Those who have had experience with pleuro-pneumonia know how difficult it is to make a diagnosis when you have only one of these animals, and no way of tracing the history of that animal. You may find the condition of the lungs, which resembles pleuro-pheumonia, but which also resembles other diseases. Wc all know that pleuro-pneumonia produces a peculiar effect to the appearance of the affected lungs; but those peculiarities are also found in lungs which have received their irritation from other germs and sometimes by other agents. So that it can hardly be claimed to-day that these symptoms are always those of contagious pleuro-pneumonia. Of course, when you take those symptoms of pleuro-pneumonia and couple them with other characteristics which we generally see, then we begin to have very positive evidence of the diagnosis. At any rate, it seemed to us there might be an error in diagnosis, and on the other hand it was possible that there might be pleuro-pneumonia going abroad from our country from our cattle shipped from sections of our country where we did not know the disease existed. It was in the West before it was found, and it is just as possible it is out here somewhere as it was then, although, if it had been here for any length of time we would have known it. Yet there is always a possibility, and for that reason it has seemed best to have our representatives on the other side.

It may be said that the British inspectors do not claim to have found any contagious pleuro-pneumonia among our cattle since February last, and since that great progress has been made in the eradication of the disease on this side. The fact is, there is very little pleuro-pneumonia in this country. I may almost say we have had none for the last six months, and as soon as it is found here the animals have been slaughtered and the disease eradicated. But in this country, as in other countries, the disease continues to appear in certain places. One reason for its re-appearance was indicated to you in the views which I showed here this morning, that stables which have been infected were retained with the infection in them, and in some of those cases it was not until the places had been utterly destroyed, the soil dug up and thoroughly disinfected by saturating the soil with corrosive sublimate. that we have finally succeeded in getting rid of the disease. After such a treatment we have had no second outbreak. But it takes time to find these different places where the disease lurks, and it also takes time to get all the old chronic cases, and that is why the disease still continues to re-appear. We have only had very few cases in Brooklyn, so that it hardly seems we could have exported the disease.

That is about the status of the matter as it is at present. England, of course, wants to avoid any importation of the disease. We hope, of course, that by showing a clear bill of health on this side, that there is no danger from pleuro-pneumonia by shipping our cattle over there. England wnats our cattle for several reasons, because of its economy among others. This is an important matter to our people, and if they can be made to understand the good work which the veterinary surgeon has done for the shippers in this country, the time may come when they will fully appreciate it.

Dr. Weber—I would like to know what is the probability of our importing the disease from England to this country?

Dr. Salmon—That is putting the shoe on the other foot. England has a good deal of pleuro-pneumonia in Ireland and in Scotland. They have been working on it over there for quite a number of years without making much progress. At the last session of Parliament they secured a new law which transferred authority in cases of pleuro-pneumonia from the local authorities to the general authority in London. Now they hope to go ahead and get the disease eradicated.

We have our quarantines against the cattle from England, and will, of course, maintain them until they show a clear bill of health. I do not think there is much probability of importing the disease in cattle that come through our quarantine stations, yet there is a bare possibility that the cattle may contain the infection, although if our officials are alert it would be almost impossible for the disease to escape detection.

I find those who know most about pleuro-pneumonia are the most conservative concerning our ability to absolutely wipe out the disease. It was said to me by one of the highest officials of the British Government that if they succeeded in ridding the country of pleuro-pneumonia in six years, or double the length of time it required in this country, they should be happy.

Dr. Baker—Do our inspectors inspect the animals before or after they are killed?

Dr. Salmon—All American cattle landed in Great Britain are slaughtered on the docks within ten days after being landed. Our inspectors are very careful to examine, as far as possible, the American cattle as they go off the ship, and they have the promise of the British inspectors that in case they find anything which they consider to be pleuro-pneumonia, it will be brought to the attention of our inspectors at once, and they will be given every opportunity to examine the cases and submit their report to our government. Our inspectors do not see the cattle killed. It is absolutely impossible for any one man, at any one of these places, to see the cattle killed; there are too many of them. There is a string of slaughterhouses a half a mile long in Bedford, where they are killing cattle all the time during the greater part of the day. A man might watch two or three houses but he could not watch them all. Probably it would take fifty men to see the organs of all the cattle slaughtered. At Bedford, some of the wards are immense institutions. I was surprised to see what permanent buildings they had erected; we haven't anything in this country to compare with it.

Dr. Baker—Another point I would like to have brought out is whether the English government require all animals to be inspected after slaughtering, or, in cases of disease, are they dependent upon the courtesy and honesty of the British butcher to report it.

Dr. Salmon—As I said before, their inspectors do not see the cattle slaughtered. Of course, if they find things which they are unable to detect during the life of the animal they must depend on the butchers. What arrangements they have, or how much they can depend upon them I cannot say.

Dr. Liautard—Do the American and English cattle go to these same wharves? There are cattle from other parts of Europe there, and there might be a possibility of the American cattle mingling with cattle from Holland and possibly from other parts of the country.

Dr. Baker—Under those circumstances it is possible that pleuro-pneumonia may be taken there by these continental cattle and the trouble ascribed to the shipments from America.

D. Salmon—That is possible, but I do not think very likely. The great bulk of our cattle going to Great Britain are run into pens by themselves, and they are handled by a class of men entirely different from those who handle continental cattle. I do not believe there is much possibility of their being mixed. They are kept separate until slaughtered, and until slaughtered, those who handle them know where the cattle come from; and I think those who handle them are rather prejudiced in our favor than against us. I do not think any mistake of the kind referred to has occurred.

D. Clement—I was very much interested in the paper of Dr. Schwartzkopff. Having had some experience, I know that a man with his training makes him an authority such as none of us would pretend to be on the subject. Germany is far ahead of any country in the world in sanitary science. Certainly its abattoirs are far superior to anything in this country, France or England. In Berlin, I believe, he said a great number of inspectors are employed, and undoubtedly one trained in such an institution gains information which is hardly possibly in this country. The abattoir system, however, is absolutely necessary, in my opinion, to a proper conduct of a system of inspection. Unless we can have abattoirs, it is hardly of any use to have a system of inspection of the food supply in our city. The classification which he adopts, however, I do not quite understand. If I remember right, he classifies the disease under three heads. First, he puts swine plague, and second, tuberculosis and others. Of the first crass of diseases he says that the carcasses should be totally destroyed. Of the second, he says only the part affected should be destroyed. Now, it seems to us that those diseases which are not directly communicable to man are less dangerous as articles of food than those diseases which are directly communicable to man; therefore, I do not see why those which are classified under the first head for total destruction are placed there, while those of the second class only a part of the carcass is destroyed. If I remember right, he speaks of the classification referring to the temperature of the animal to a certain extent, before death. Undoubtedly a high temperature is very obnoxious, but it is certainly not as injurious, in the opinion of many of us, as would be the eating of the flesh of animals containing germs of diseases which are directly communicable to man. I would like to ask him to kindly explain again the basis of his classification of those diseases which he includes under the several heads.

Dr. Schwartzkopff—The question of Dr. Clement is correct. The diseases were classed under three heads: First, such as are prohibited by ordinary police regulation. Some they are not allowed to slaughter at all in Germany and Austria and parts of Switzerland, and I think, in Italy, although I am not much acquainted with such laws. Prof. Liautard will understand that France has no such laws, neither has England or Russia.

Dr. Liautard—Yes; France has the same laws.

Dr. Schwartzkopff—Yes; all over the continent those laws prevail, with the exception of Russia and in England.

Under the second head I have classed those diseases where slaughtering is permitted, to ascertain whether the whole or part is fit for human food to be used for industrial purposes or to be absolutely destroyed. Some parts of the animal may be given for consumption, or may be used in the rendering tanks or be totally destroyed. There are three different doors which are open to the sanitarium. As far as tuberculosis is concerned, we all know that animals in which tuberculosis is found should be destroyed, that is, from a theoretical standpoint. But we all know that in cattle shipped from several sections of the country, there will be found animals more or less affected with tuberculosis, some of them in such a small degree that although they show tuberculosis, otherwise the animal is in a perfect condition for food. I personally adopt the views of this country; but so far as Germany is concerned, from which I largely adopt this view, the German authority on these sanitary matters allow the use of cattle diseased with tuberculosis, for instance, if in one part of the lung. As soon as tuberculosis is shown, which is very easily recognized by the affection of the lymphatic glands and through the body, it is generally considered that the animal is unfit for food, and it is so, without doubt.

Dr. Clement—I wanted to get at the basis of your classification. I quite agree with you that cattle affected with tuberculosis in a small degree are not injurious as an article of food.

Dr. Schwartzkopff—Excuse me; I should have said, of course, that the diseased parts are destroyed.

Dr. Clement—It seems to me that a little too much stress may be laid upon some of the other diseases which render the animals unfit for food. I do not think it will do any harm, for instance, to eat pork from a pig which died of hog cholera, even though the animal was very sick. I would like to ask Dr. Schwartzkopff if diseased meat is sold in Germany and so specified? If, from an economic standpoint, they do not condemn the whole animal, but pass it as diseased and sell it to the people who know what they are buying and take their chances?

Dr. Schwartzkopff—Dr. Clement is right. There are laws in Southern Germany which provide for a classification of the meat. In the first place, perfectly sound meat for the market. Then there will be given meat, for instance, from animals with the swine plague, which are known not to injure human beings, but which rather from feeling that such meat is not from perfectly sound animals, persons would rather not eat. Such meat is classified and sold cheap. Not under a general law, but local ordinances of the municipality, from what you would call here aldermen. [Applause.] These are simply ordinances, which allow this meat to be sold to the poorer classes. In this country you will hardly like that very much. In America the citizen is a much stronger person than in most of the countries of Europe, with the possible exception, perhaps, of England. Here all classes of people want good, sound meat, and nothing else. It will take a long time in this country to establish such a law, as it has taken a hundred years in Germany to bring it about.

Dr. Williams—The two diseases of actinomykosis and tuberculosis are subjects of importance to us in Illinois. Dr Clement has said it was impossible to condemn all these animals; Illinois has found it possible to condemn them, and has done it very successfully. We have some lively fights on hand on the subject of actinomykosis. We probably have more animals affected with this disease than any other part of the country. We have had, for instance, in one series of cattle sheds here about one hundred cases of actinomykosis, and we had quite a little trouble right here in Chicago on that subject.

Dr. Atkinson—It occurs to me, in considering this subject, there is one feature lost sight of. It has always seemed to me that while an animal was on its four feet it was a fit subject for the veterinarian, but when hanging up by the quarters for food, it was more properly a subject for the board of health to deal with. I have had some experience. Five years ago I was appointed State veterinarian of Wisconsin, and was somewhat enthusiastic, as all beginners are. Among the first cases I met was a party that had a drove of 200 hogs, nearly ready for the market, in which hog cholera had made its appearance. I happened to be visiting in the neighborhood; he heard I was coming and he attempted to ship them. I succeeded in getting him to take them back to his farm. The old man protested that it meant ruin if he had to lose those hogs. He said he knew if he could get them in to the Chicago market he could realize on them. I expected I would be sustained by the State board of health. The law provided that I should co-operate with them. As soon as I got home I notified the Secretary of the State Board of Health, and some six or eight months afterward I asked him if he got my letter. He said, "Yes; we took it up at our last meeting." I said, "What of it?" "Well," he said, "there is nothing to show that hog cholera does people any harm, and we concluded it was not a matter for our board to interfere with." Now, how far would I have been sustained had I gone on and acted in that matter? I do not believe veterinarians can act alone. I had another experience almost similar. This subject of tuberculosis has attracted a great deal of attention. It was made the subject of discussion in a meeting of veterinarians, sanitarians and board of health officers, held at Springfield last fall, and the question of how far our jurisdiction would go was brought up by myself at that time, as it is now. We called in the Secretary of the State Board of Health, and asked him what his opinion was. He said it was not established that they were contagious diseases. He said there was a theory advanced that they were, but it was not generally accepted in the medical profession that they were. If the veterinary profession is going to place itself on record as saying that that fact is established—that they are contagious diseases—I believe there is some doubt whether tuberculosis is a disease or not, but in the other there are some diseases transmissible from animal to animal and to mankind. This is a wide subject, and any attempt at securing legislation, it seems to me, would be more properly attempted when supported by the board of health after we have reached that point. It may be that the veterinarian has better fields for observation, but for my own part, I have always looked at the medical profession as one of the sources from which we can gather information in that direction.

Dr. Schwartzkopff—These remarks are very interesting, but I think this is one of the most profitable fields of work for the veterinarian.

As to the question of tuberculosis, as I said before, theoretically it is not contagious. Some time ago there was a dispute going on, and I expressed my views, which, I am sorry, were contrary to the belief of most of you in this country. Personally, I like to go where you go; I do not want to put myself on record as against you or your ideas, but when I am forced to do so I must be candid, and say I do not believe it is contagious, and I base my opinion on my own experience, as well as my theoretical studies, in handling cattle in the Berlin slaughter-houses. As long as you are dealing with the living animal it will be always difficult to decide some of these questions. The only way it can be done in many cases is by post-mortem, to determine whether the meat is fit for human food or not. There may be some cases in which the meat of the animals would be dangerous, and in others where it is not. This is a question of importance, and you can be sure that you will be compelled in the next few years to form your opinions on this matter.

Dr. Salmon—This is a question I am considerably interested in, and I am glad it has come up. I only wish the association would make it a special subject for discussion at the next meeting. I would like to see the members come in prepared to present their views and criticize the opinions of others, and thus bring out the truth, as it is always brought out, by conflict of opinion. It is only by discussion and a comparison of notes, and by the interchange of ideas, that we finally sift the wheat from the chaff, and get down to the truth about a matter. Meat inspection is a matter that it will not be many years before it will prevail in every locality in the country. It is a national question, and a very important one. As a profession and as members of the National Veterinary Assiciation we should have clear ideas upon the subject, and be ready to give reasons for the belief that is within us. I think some members of our profession have been rather hasty in arriving at conclusions. I believe the inspection should only condemn the animal in case the red flesh is affected, and in case the tuberculosis is disseminated throughout the body, some claim. They claim if the lung is affected the flesh of the animal is safe. Now, is that a safe conclusion for us to reach. Have all of us reached the point where we can say there is no possibility of danger in consuming the meat of an animal that has tuberculosis in the lung. If it is true that these germs circulate in the glands and in the udder, why don't they circulate in the meat that is used for human food? I say it is too early for us to say that the meat of an animal is safe for human food because we are unable to find the germs of these diseases in the lean meat. If we find these germs in the blood, why do we not find them in the red flesh? If they are in some parts, they must have circulated in different parts of the body. How do we know how far they go, and how do we know that they are not in all parts of the carcass, having circulated through it?

As to the diseases which are contagious, there is strong feeling against the use for food of animals so diseased. It is a very hard to draw the line. Sometimes you have a case of acute pleuro-pneumonia. You do not know the temperature, you do not have the temperature raised high enough so that you can say there is any rise in temperature. Who can say that there are not poisonous elements all through the body, which may be very injurious to people partaking of the flesh?

There are a great many facts to be taken into consideration on this question of meat inspection, and I hope before the next meeting our observations will be made thorough. We have just entered on the threshold of our knowledge of contagious diseases, and it is too early to come to any positive opinions, especially when those opinions run rather counter to the opinions which have heretofore prevailed.

Dr. Meyers, Sr.—In regard to actinomykosis, I have been reading the slaughter-house reports from Berlin, and it appears there that the diseased part is thrown away, and the other portion allowed to be consumed. And even in tuberculosis, I believe, there is a limit of the same description. Another theory is that where only a portion is affected, it is diseased through and through. So I guess we ought to make a distinction between that which is to be consumed and that which is not to be allowed.

Dr. Hawkins—I will state what has been done in the city of Detroit. When the health officer discovers a case of actinomykosis he orders the destruction of that animal, and of course, that ends it right there.

Now with regard to the inspection of meat. In our city, to give you an idea of what our inspection is, our present meat inspector is a cigar peddler. He knows as much about healthy and unhealthy meat as a dog does about book-keeping. In Michigan, two years ago, there was introduced into the legislature a bill providing for a live stock inspector for the inspection of hogs and cattle brought into the different stock yards. The only way we have now to prevent the sale of diseased meat on our market is through the local inspector, who is appointed by the board of aldermen. If he is a good Democrat he stands a pretty good chance of getting in. That is the way our inspectors are appointed.

Dr. Baker—I do not know that there is much Democracy in contagious diseases in Illinois; but as regards the disposition of cases of actinomykosis here, as stated by Dr. Williams, we have been able to deal with it. In Illinois the officials have acted under the advice of their veterinarians, and we look upon actinomykosis as a disease which renders the animal unfit for food. In a late issue of the *North American Review* there is an interesting article on the longevity of Americans living in the central portion of the United States, and it appears that it is greater there than in any part of the world to-day. That in a great measure is due to the character of the food eaten by the inhabitants. They are more particular as to the quality of the meat and the fruit they consume, and consequently they are freer from those diseases which lessen the longevity of the race.

While we have not actually found the germs of actinomykosis, though considerable microscopic work has been done here, yet we have found them in most of the internal organs, the lung, liver and intestines, quite a considerable distance from the digestive tract. There is no question in our opinion but what it is diseased meat, and while I do not know positively that it does not exist in the muscles or the red flesh that is eaten as lean beef, the safest way is to guard against any possible contamination from that source by condemning it on general principles as diseased meat.

Dr. Faust—I think the Jewish people give us a fine illustration looking at them as a race from beginning to end. Would a Jew eat a piece of meat affected with actinomykosis?

Now then, as far as tuberculosis goes, does he eat that? I think not. I think the gentleman that just spoke expressed the true sentiment that we should keep away from danger and condemn it as unfit, destroying everything that is diseased. We have plenty of good meat in this country and don't want to eat diseased meat. If we should ever come to that point, when, owing to a dense population, we are obliged to eat diseased meat we can then consider how injurious it will be.

Dr. Williams—This question is of great importance, and it has been difficult for me to keep still. We wish to further discuss this subject, and therefore, I move that the discussion be now closed, but that a committee of three be appointed by the Chair to consider the subject of actinomykosis and tuberculosis, which committee shall submit a report at the next meeting of this association.

Dr. Clement—Would amend that motion by adding that the committee also consider the subject of meat inspection.

The motion was seconded and carried unanimously.

The Secretary announced the banquet at the Palmer House at 8 o'clock this evening.

Secretary Hoskins—I move you, Mr. President, that we extend a vote of thanks to the Western Local Committee for the preparations they have made and for the successful way in which they have carried out the details for this meeting.

Seconded. Carried unanimously.

I also move that a vote of thanks be extended to the retiring officers of 1889 and 1890, which we neglected to do on the retirement of our president who gave us such very efficient service during the last year, and who contributed so largely to the success of this meeting, the most successful we have ever held, 109 members have been in the room at one time.

Dr. Atkinson—I move to amend by extending a cordial vote of thanks to all the retiring officers as well as those who have been re-elected.

Seconded. Carried unanimously.

Dr. Adair—I move you that a vote of thanks be tendered to Dr. Salmon for the very able manner in which he presented his paper, and the instructive way in which he illustrated his ideas by the use of the lantern.

A Member—I move you that we also include a vote of thanks not only to Dr. Salmon but to Drs. Liautard, Huidekoper, Schwartzkopff, Meyers and Berns, or in other words, all those who have instructed and entertained us by their very able papers.

Seconded. Carried unanimously.

On motion duly seconded, the meeting adjourned.

Adjourned *sine die.*

*Keystone Veterinary Medical Association.*—The Keystone Veterinary Medical Association held its regular meeting at the College of Physicians, October 4th, the President, Dr. Hoskins, in the chair. Drs. Zuill, Goentner, Glass, Hoskins, Huidekoper, Kooker, Bridge, Vandegrift, Williams, Cullen, Webster, Ridge, Lusson, Formad, Magill were present.

Dr. Hoskins spoke of the small amount of work the society had done the last year. He said it was the duty of every member to do his part, and to report everything of interest to the association, and so make the meetings valuable. He spoke of those who had been absent during the last year, and recommended that when a member had been absent a whole year that his name should be stricken off the roll.

The minutes of the September meeting were read and approved.

REPORT OF TRUSTEES.

"We, the Board of Trustees of the Keystone Veterinary Medical Association, do recommend that the resignation of Thomas B. Rayner be accepted (Signed) DR. W. S. KOOKER,
DR. L. O. LUSSON.

It was moved and seconded that Dr. J. B. Rayner's resignation be accepted. Carried. Committee on legislation requested the attorneys, Messrs. Magill and Williams, to frame a bill to send to the legislature, to cover the defects of the present law. They reported a colored man as practicing without having registered. Dr. Hoskins said the man had testified in court, and thought it best not to prosecute him. Dr. Zuill proposed the name of Dr. E. H. Landes, of the Veterinary Department of the University of Pennsylvania, and Dr. Huidekoper proposed the name of Dr. M. W. Drake, American Veterinary College, for membership. Dr. Huidekoper read a paper entitled "Atmosphere as a Predisposing Cause of Disease."

Dr. Glass reported a case of hip lameness, which he blistered over a small area, but after it had been greased a few times the whole rump became blistered and the hair came off. He could not account for it. The owner thought the groom might have blistered it for spite. Dr. Zuill said rancid lard or sweet oil will blister, and that was the cause of the said condition. Dr. Huidekoper thought it might be due to lard, or might be a consecutive erythema. Drs. Magill, Williams, Ridge and Hoskins thought it due to the use of rancid material.

*Report of Committee on Tumors.*—Dr. Formad had a few sections of the tumor, and the tumor itself, from a dog's neck, which had been placed under discussion at previous meetings. He says it is a sarcoma. He made some remarks on tumor and the manner to get sections for the microscope; they should be taken from deep in the tumor. He recalled a case where a superficial examination of a tumor caused the diagnosis of a sarcoma, and, on this ground, a dangerous operation was performed, resulting in the patient's death. A more thorough examination revealed only the existence of a lipoma. The section had been taken from an ulcerating surface. Dr. Ridge gave the history of the said tumor, which contained a cyst, with fluid.

The President stated he would from time to time request from members special information that they were fitted, by their work and position, to give.

He appointed as committee on essays Dr. Magill, chairman, and Dr. Ridge. The president referred to the existence of a number of delinquents on the Treasurer's book, and further directed that the Treasurer render an account to each member in arrears at least once every six months.

Dr. Goentner moved that Dr. C. Schaufler be expelled from the association for non-payment of dues. Carried. The Secretary was directed to notify him of the same. Bill of Dr. Ridge, secretary for last year, was acted on, and Treasurer instructed to pay him. Dr. Ridge suggested that one or two members should hold papers in reserve to replace absent essayists. Dr. Zuill rose to speak of a violation of the code of ethics by one of the members. The President called him to order, and asked him to place the charge in writing, and it would be properly referred to the board of trustees. Adjourned.

C. H. MAGILL, *Secretary.*

*Massachusetts Veterinary Association.*—The regular meeting was held at 19 Boylston Place, Boston, September 24th, 1890, President Thomas Blackwood in the chair. Members present: Drs. Blackwood, Howard, Osgood, J. S. Saunders, Winchester, Marshall, Ferguson, Peterson, Hadock and the secretary; honorary member, Dr. Stickney; guest, Dr. E. C. Beckett; essayist, Dr. Emerson.

After transacting routine business, a paper by Dr. Emerson was read, on "Punctured Wounds of the Foot, Involving the Joint," stating that the most serious are those where the flexor tendon, the synovial sac, or the bony articulation are injured. He considered them also grave, as they are of such common occurrence that many owners and drivers of horses, and not a few blacksmiths, have an infallible quick "cure" of their own, which they apply in all cases without regard to location or extent of injury, and more than half the cases that the veterinarian is called to, have been treated in one way or another for from one to four or five days. When the owner finds that the animal is constantly growing worse, he sends for the veterinarian and tells him what he has done, and how many times he has done it before, without ever losing the use of the animal for a day. In the locality of the joint it is never advisable to probe deeply unless synovia is already discharging from the opening. Dr. Emerson cited the clinical symptoms of a number of cases. He uses injections of carbolic acid, aconite as a febribuge, and opium and camphor as anodynes.

Dr. Ferguson asked the essayist how he accounted for the emaciation being more rapid following wounds of the hind feet than those of the fore-feet. The essayist thought that it might be because it is harder for a horse to stand upon two fore-feet and a hind foot than it is to stand upon two hind feet and one fore-foot.

Dr. Howard thought that next to a case of colic at 2 o'clock in the morning, that he knew of nothing that would make a man more tired than a badly punctured wound of the foot. He said he wanted to see it as soon after it occurred as possible, and to be sure and pare it off enough. He also spoke of a plan of treatment suggested to him by a certain irregular practitioner of Boston, for horses which had lost the use of a foot, which he thought a very brilliant plan. It consisted of a crutch, which this man claims to have invented; it can be fastened on the lame leg and the horse turned out to pasture.

Dr. Marshall spoke of the application of Dr. Bern's pads to punctured wounds of the feet, as he had seen them used by the inventor during a recent visit to Brooklyn. The pad is first wet in a solution of fenol and kept in place by a bandage ; the foot can also be soaked in a pail or tub containing a solution of the fenol during the day.

Dr. Peterson likes to cleanse the wound daily with a solution of corrosive sublimate applied with a syringe.

Dr. Beckett said he used a strong solution of carbolic acid, 85 per cent. to 90 per cent., made soluble by adding glycerine ; it seems to have a powerful effect in stimulating the injured parts to heal when applied early.

The President also spoke of the use of carbolic acid ; in one of the car stables under his charge it is customary to pull the nail out after it is picked up, and then to pour a few drops of pure carbolic acid into the nail hole at once, and often with very good results. He did not approve of paring off too much horn at first in these cases, as it was not always necessary.

Dr. Howard said that he had often obtained very good results on many kinds of wounds on various parts of the animal by painting the part with an 85 per cent. to 90 per cent. solution of carbolic acid applied by means of a glass rod.

Dr. Howard moved that a vote of thanks be given the essayist for his paper, and that the Secretary cast one ballot for his admission as a member of the association. Seconded by Dr. Marshall. Carried. Dr. Emerson is elected a member.

Dr. Stickney spoke of a case where a horse picked up a screwdriver without a handle, which made a good and speedy recovery.

At the request of the President, Dr. Winchester then gave a short account of his visit to Chicago and the meeting of the United States Veterinary Medical Associotion, the 16th and 17th instants.

Dr. Osgood brought up the subject of rabies by reporting a suspicious case of his at Springfield, the dog being in a cell at the police station under observation ; he had bitten two children, but it was still uncertain whether he was really rabid or only vicious. This led to quite a discussion upon rabies, the President, Drs. Winchester, Stickney, Ferguson, Beckett, Peterson and the secretary taking part. The conversation then turned to paralysis of the muscles of the lips, face, nostrils and eyelids in horses ; the President, Drs. Hadock, Peterson, Beckett and the secretary citing cases which had occurred in their practice. Meeting then ad!ourned.

AUSTIN PETERS, *Secretary.*

